# *N*-Nitrosomorpholine-induced oncocytic transformation in rat endocrine organs

**DOI:** 10.1186/s40001-024-01654-5

**Published:** 2024-01-20

**Authors:** Emre Gezer, Cüneyt Özer, Turgay Şimşek, Büşra Yaprak Bayrak, Gupse Turan, Berrin Çetinarslan, Alev Selek, Zeynep Cantürk, Mehmet Sözen, Damla Köksalan

**Affiliations:** 1 Division of Endocrinology and Metabolism, Darica Farabi Training and Research Hospital, Kocaeli, Turkey; 2 Experimental Medicine Research and Application Unit, Kocaeli University, Kocaeli, Turkey; 3 Department of General Surgery, Faculty of Medicine, Kocaeli University, Kocaeli, Turkey; 4 Department of Pathology, Faculty of Medicine, Kocaeli University, Kocaeli, Turkey; 5 Department of Endocrinology and Metabolism, Faculty of Medicine, Kocaeli University, Kocaeli, Turkey

**Keywords:** Nitroso compounds, Oncocyte, Endocrine disrupting chemical, Endocrine disruptors, Endocrine gland neoplasms

## Abstract

**Background:**

*N*-Nitrosomorpholine (NMO) is one of the most common *N*-nitroso compounds. An oncocytic transformation has been demonstrated in renal tubules of NMO-treated rats. In our study, we aimed to investigate the potential transformation of oncocytic cells in 6 endocrine organs, i.e., thyroid, adrenal and pituitary glands, pancreas, testis, and bone, of NMO-exposed rats.

**Methods:**

Thirty male rats were born and raised. Fifteen of them were given a single dose of 320 mg NMO per kg body weight, dissolved in drinking water, by a gavage tube. At the end of 52 weeks, the animals in both series were killed. Right after the killing, 6 different endocrine organs (hypophysis, thyroid, pancreas, adrenal gland, bone [femur], and testicles) of each animal were excised.

**Results:**

There was no evidence of oncocytic cell development in the control group. In contrast, oncocytes were observed in 8 out of 13 NMO-treated rats: 2 in the adrenal sections, 1 in the thyroid sections, 3 in the pituitary sections, and 2 in the pancreas sections. Thesticle and bone sections were completely normal.

**Conclusions:**

We showed that NMO induced an oncocytic change in pancreas, thyroid, pituitary, and adrenal glands. To date, no identified specific environmental risk factors that lead to an oncocytic transformation in endocrine glands have been reported previously. Given the increasing prevalence of endocrine-disrupting chemicals in the environment, personal care products, manufactured goods, and food sources, there is a need to advance our understanding of the pathological mechanisms underlying oncocytosis in endocrine organs.

## Background

*N*-Nitroso compounds have become a potentially large source of exposure for human beings to chemical carcinogens in the recent years. *N*-Nitrosomorpholine (NMO) is one of the most common *N*-nitroso compounds, which has been frequently detected in the discharged water from sewage treatment plants, some cosmetics and toiletries, fine-cut chewing tobacco used for snuff-dipping and as an air pollutant in regions where tire-manufacturing and rubber factories are located [1–4]. This tumorigenic compound has been shown previously to be mainly hepatotoxic, inducing hepatocellular carcinoma [5, 6]. Moreover, NMO also triggered the development of neoplasms in the respiratory and upper digestive tracts, thyroid gland, brain, and spleen in various animal experiments [7–10].

An oncocyte is an enlarged cell with abundant eosinophilic, granular cytoplasm, in which a large hyperchromatic nucleus exists [11]. This oncocytic change is caused by proliferation and accumulation of mitochondria within the cell, and it has been mainly attributed to the alterations related to the mitochondrial DNA [12]. These mitochondrial anomalies are triggered by chronic inflammation secondary to aging or various chemical carcinogens such as NMO [13–15]. Various studies have demonstrated an oncocytic transformation in renal tubules of NMO-treated rats [15–20]. In addition to that, germline mutations of the *Gene associated with Retinoid-Interferon-induced Mortality (GRIM)-19* have also been indicated as a possible etiological factor in the development of an oncocytic tumor [21]. This metaplastic differentiation has been observed in various organs, such as thyroid, parathyroid, pituitary and adrenal glands, pancreas, kidney and paraganglia [13]. In our study, we aimed to investigate the potential transformation of oncocytic cells in six endocrine organs, i.e., thyroid, adrenal and pituitary glands, pancreas, testis, and bone, of NMO-exposed rats.

## Materials and methods

### Characterization of N-nitrosomorpholine (NMO)

NMO was provided from MedChemExpress, New Jersey, USA; via a distributor located in our region, named Suarge Biotechnology Co., Ltd., Istanbul, Turkey. The purity and solubility of NMO were reported as 98.22% and ≥ 100 mg/mL at 19 ℃, respectively. The appearance was solid < 29 ℃ and liquid ≥ 29 ℃. The chemical could be stored at 4 ℃ for 2 years. It was also reported that NMO was light-sensitive and could be stable at room temperature for more than 14 days in neutral form in dark.

### Animal experiments

Thirty male Sprague–Dawley rats (about 8-week old) weighing about 200 g were born and raised in the Experimental Medicine Research and Application Unit of our institution. Fifteen rats were given a single dose of 320 mg NMO per kg body weight, dissolved in drinking water, by a gavage tube. The amount of this chemical agent was determined according to a previously reported rat study, in which a single dose of 320 mg/kg NMO was administered to the animals [20]. The animals were placed in different cages in groups of 5 and maintained under constant conditions (20 ± 2  C and 12 h light/dark cycle). The rest 15 rats, used as controls, were also administered placebo by gavage in the same day and housed in groups of 5 in the same room with the others. Afterward, the rats were kept on a standard laboratory diet and tap water for 52 weeks (*ad-libitum*).

The maintenance period from the start of the experiment to the killing was based on various rat studies which revealed data on the time of appearance and incidence of oncocytic lesions [10, 14, 17, 18]. Laboratory animals were cared for and fed in Eurostandard type IV cages (1354G EUROSTANDARD TYPE IV 598 × 380 × 200 mm, floor area: 1820 cm^2^) defined under the condition of “management and implementation instructions for the breeding and protection of animals used for experimental and other scientific parts”. Only health monitoring protocol was to make a daily inspection of rats’ behavior and record in case of any abnormality. No abnormal behavior was observed, even in the rats which were deceased during the experiment. In addition to that, observation of daily food and water consumption of the rats was made and no differentiation of any rat’s consumption from the others was observed.

At the end of 52 weeks, the animals in both series were killed using xylazine and ketamine intraperitoneal injections. All rats were killed between 9 a.m. and 1 p.m. in 2 consecutive days; NMO-treated group in the first day and controls in the second day. The main reason to kill 2 groups in 2 consecutive days within the similar daytime period was to control for the circadian rhythm differences between the groups. Right after the killing, six different endocrine organs (hypophysis, thyroid, pancreas, adrenal gland, bone [femur], and testicles) of each animal were excised. The only excluding criterion in this study was the development of mortality during the experiment.

### Histopathological examination

Each organ was stored rapidly in a plastic sterile container full of 10% formaldehyde solution. Thereafter, tissue samples taken from the removed organs were fixed and embedded in paraffin for light microscopic examinations. Each of the organs was examined by serial sectioning. Section thicknesses were 3 µm. Nine separate sections were made for each organ. The histological sections were stained with hematoxylin and eosin (H&E). The presence of oncocytic cells in each tissue section was investigated under the microscope by two pathologists. The 1st, 5^th^, and 9th sections were examined. Presence of any oncocytic cell was expressed as percentage. The average of the oncocytic cell percentages in these three sections for each organ was recorded as the oncocytic cell percentage specific to that organ.

## Results

### Mortality data

A total of three rats, two from the carcinogen-treated group and one from controls, were found deceased within this period, by the weeks 44, 50, and 48, respectively, after the beginning of the experiment, respectively. Diagnostic necropsy examinations of the deceased rats revealed normal postmortem changes; thus, no additional examination was performed.

### Histopathological outcome

Six endocrine organs (hypophysis, thyroid, pancreas, adrenal gland, bone, and testicles) of 27 rats (13 NMO-treated and 14 controls) were evaluated in a total of 162 H&E stained sections.

**Fig. 1:**
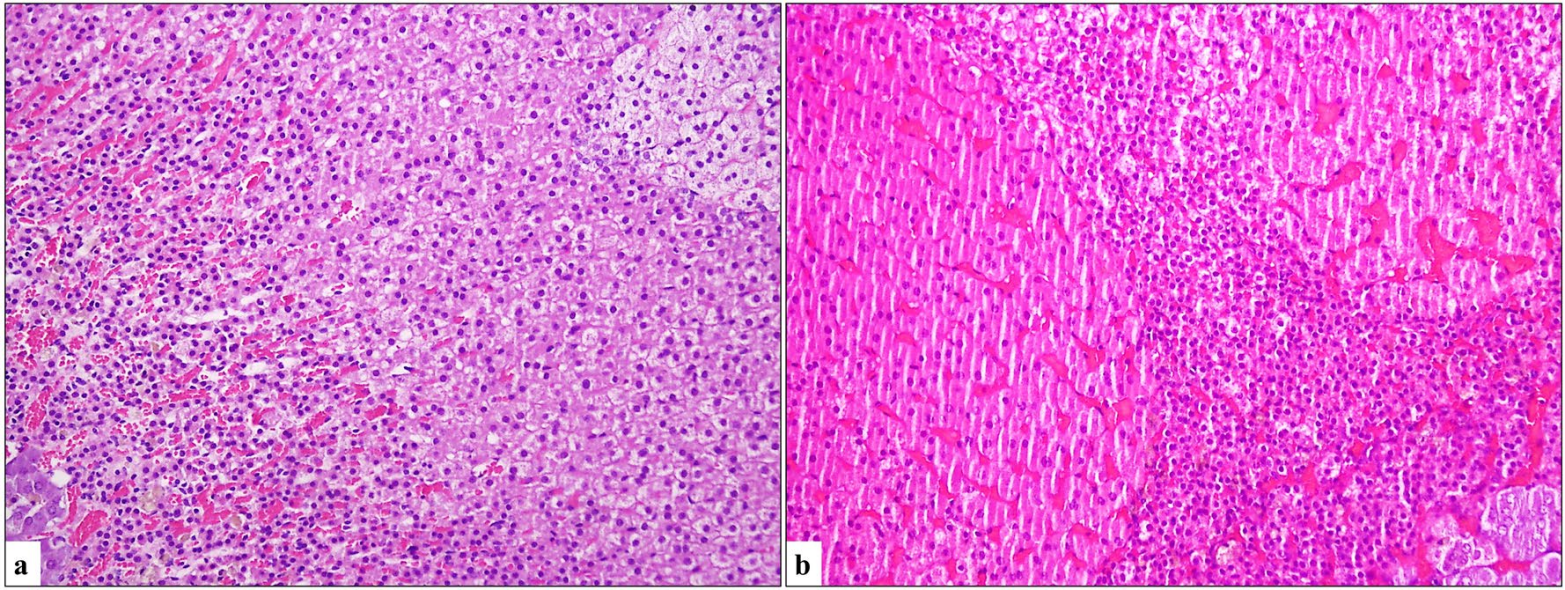
**a** Normal adrenal gland layers (inner to outer layer) (H&E, × 200). **b** Oncocytic cell groups with well-defined cell borders, deeply eosinophilic, granular cytoplasm, small round nuclei (H&E, × 200)

**Fig. 2:**
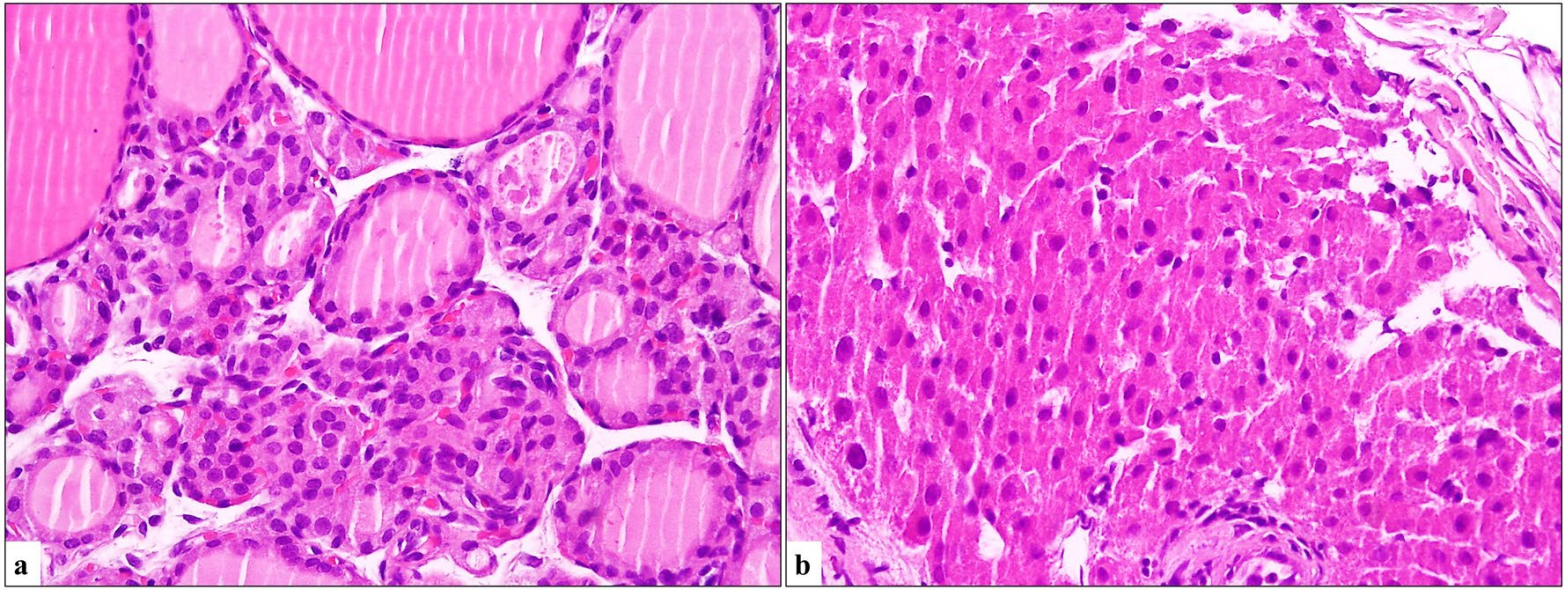
**a** Thyroid parenchyma composed of normal follicle epithelial cells (H&E, × 400). **b** Oncocytes with large size, prominent cell borders, eosinophilic, and granular cytoplasm on thyroid sections (H&E, × 400)

**Fig. 3:**
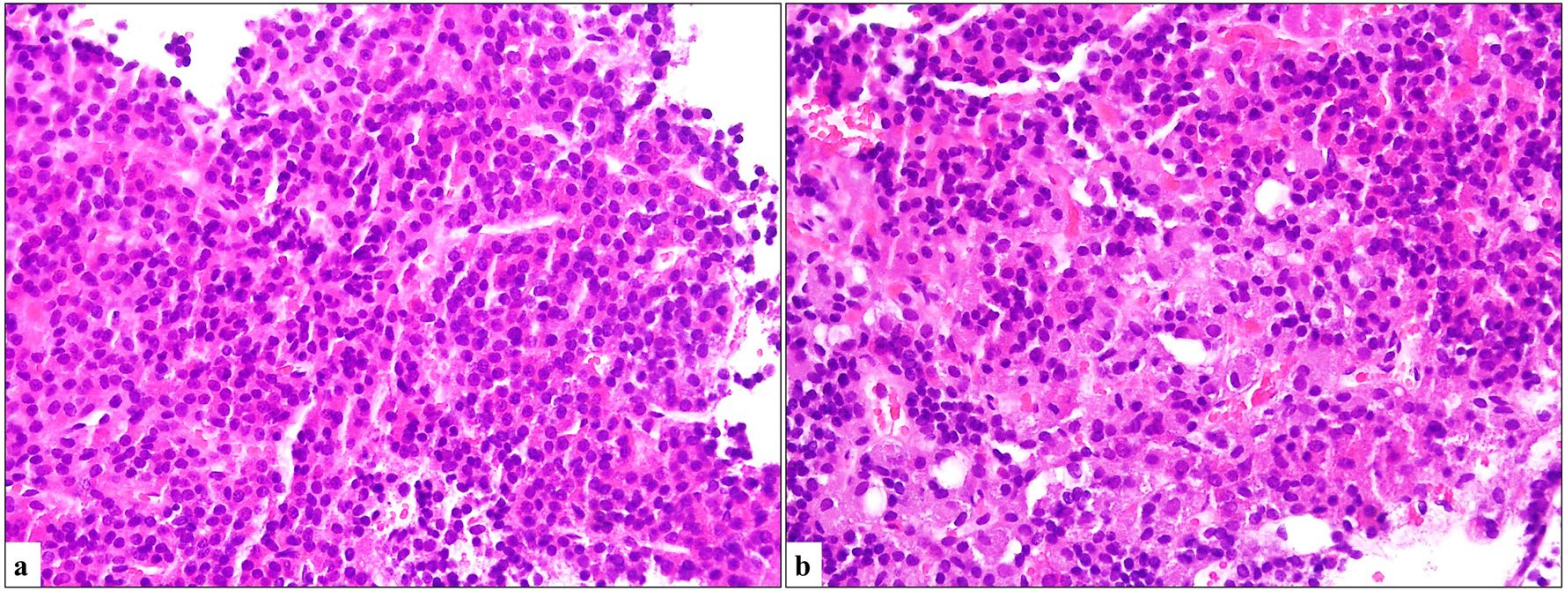
**a** Normal pituitary tissue showing relative cellular monomorphism with a single cell type (H&E, × 400). **b** Oncocytic cells scattered in the pituitary parenchyma (H&E, × 400)

**Fig. 4:**
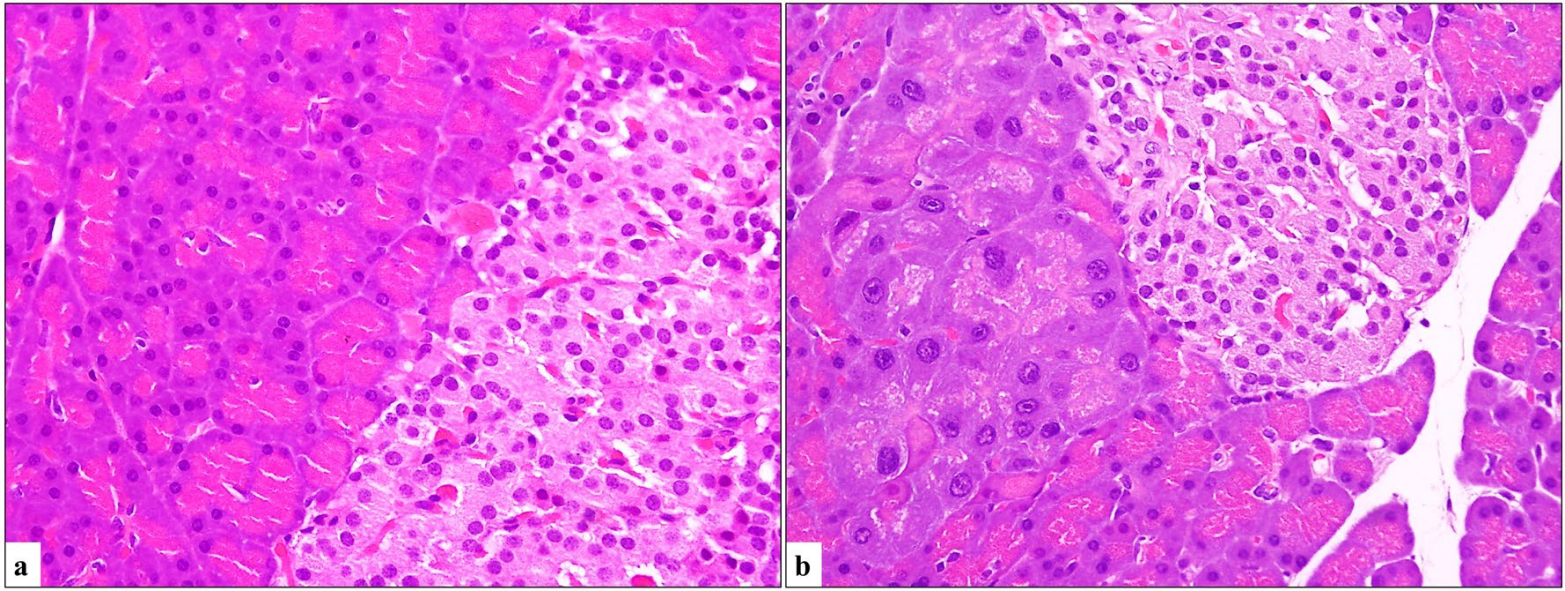
**a** Normal pancreatic parenchyma (H&E, × 400). **b** Oncocytic cells located between acinar cells and endocrine cells (H&E, × 400)

There was no evidence of oncocytic cell development in the control group. In contrast, oncocytes were observed in 8 out of 13 NMO-treated rats: 2 in the adrenal sections (Fig. 1), 1 in the thyroid sections (Fig. 2), 3 in the pituitary sections (Fig. 3), and 2 in the pancreas sections (Fig. 4).

Interestingly, of these 8 animals, each one developed oncocytic transformation in only one organ. The percentages of the oncocytes in the sections are described in detail in Table 1. The cells in the testicle and bone sections were completely normal, with no oncocytic appearance.

**Table 1:** The percentages of oncocytic cells in the sections of the endocrine organs of the NMO-treated rats

Rat #	Percentages of oncocytes					
	Adrenal	Thyroid	Pituitary	Pancreas	Bone	Testis
1	10	–	–	–	–	–
2	10	–	–	–	–	–
3	–	–	–	–	–	–
4	–	–	–	–	–	–
5	–	–	10	–	–	–
6	–	–	20	–	–	–
7	–	–	–	–	–	–
8	–	–	–	5	–	–
9	–	–	–	5	–	–
10	–	20	–	–	–	–
11	–	–	10	–	–	–
12	–	–	–	–	–	–
13	–	–	–	–	–	–

## Discussion

The results of the present study showed that NMO is a chemical compound which induces oncocytic transformation in the rat endocrine organs, i.e., adrenal, thyroid and pituitary glands, and pancreas. To date, in terms of oncocytic change, rat kidney has been the only proven organ which developed oncocytic tubules and oncocytoma after exposure to NMO in various animal studies. Similar to the methods of our study, Nogueira et al. administered single doses of 320 mg/kg of NMO by a gavage tube to a group of rats and started killing the rats 50 weeks after the start of the experiment [20]. Besides this group, three other groups of rats were given NMO in single doses of 200 mg per body weight dissolved in the drinking water at concentrations of 50 mg/100 ml or 20 mg/100 ml for 7 or 3 weeks. By the week 50 after the start of NMO administration, renal clear cell tumors and/or tubules were most frequently observed in the group which was treated with 320 mg of NMO per body weight and the authors reported that the granular acidophilic cells with larger central nuclei were more prevalent than clear cell lesions in all sections. Previous studies reported the appearance of oncocytic renal tubules after 15 and 23 weeks’ exposure to NMO [15, 16]. In our study, we aimed to increase the likelihood of developing oncocytic lesions in the endocrine organs by maintaining the rats for 52 weeks.

The cytoplasm of an oncocyte is described as “swollen” which is due to mainly the presence of numerous abnormal mitochondria in the cellular cytoplasm [22, 23]. The main mechanism for this granular, large, and eosinophilic cytoplasm filled with mitochondria has been indicated as the impairments in the mitochondrial DNA, which is supposed to be more susceptible than nuclear DNA to environmental carcinogens [12]. The accumulation of mitochondria has been attributed to the compensatory mechanism following exposure to toxic substances.

In our study, pituitary gland was clearly the most affected endocrine gland by the administration of NMO, in terms of oncocytic transformation. The well-vascularization of pituitary gland by the hypothalamothypophyseal portal system may be a possible explanation for this outcome, by making the gland tissue more sensitive to the environmental endocrine disruptors [24]. Oncocytic change has been mainly demonstrated in gonadotroph adenomas. A hundred cases with gonadotroph adenomas were analyzed by Young et al. and a patchy acidophilic appearance corresponding to oncocytic change was reported for most of the specimens [25]. In another study, oncocytic adenomas were described as clinically inactive pituitary adenomas, together with null cell adenomas and gonadotroph adenomas [26]. In short, the asymptomatic nature of oncocytic pituitary adenomas may cause an underestimation of the actual prevalence of pituitary adenomas with oncocytic features, especially for the areas with elevated *N*-nitroso compounds in the air and/or drinking water.

Oncocytic transformation is not an uncommon pathological finding in both benign and malignant thyroid lesions. In a recent review, the oncocytic change in thyroid pathology has been discussed in detail and it has been described in thyroiditis, thyroid follicular adenomas, follicular-patterned differentiated thyroid carcinomas, papillary carcinomas, poorly differentiated carcinomas, and medullary thyroid carcinomas [13]. In line with the literature, our study demonstrated that NMO, a tumorigenic chemical, induced an oncocytic transformation in the thyroid tissue of one rat with a high percentage (20%) of oncocytes.

In various reviews and case reports, oncocytic neoplasms have been described for both adrenal gland and pancreas [27–30]. Intraductal oncocytic papillary neoplasms (IOPNs), the precursor lesions of invasive carcinoma, are generally cystic neoplasms, which may cause pancreatic ductal obstruction. In contrast, oncocytic neoplasms arising in the adrenal glands are mostly benign and nonfunctional tumors, which rarely transform to oncocytic adrenocortical carcinoma. There are limited data on genetic mutations in IOPNs and adrenal oncocytic neoplasms. In the present study, oncocytes were observed in the adrenal glands of two rats and in the pancreas tissues from another two rats with relatively low percentages of oncocytes (5%) compared to the adrenal glands (10%). To date, there has been no report which described any oncocytic change in bone and testicular tissues. In parallel with the literature, we observed no oncocytic transformation in the sections of the bone and testicular tissues from the NMO-treated rats.

Our study has both a limitation and strength. The limitation was the lack of histopathological evaluation of those three rats’ organs. Even though those three deaths were unexpected during the experiment, the histopathological evaluation could not be performed, because the rats were found dead during daily observation, but not at the time of death; thus, the organs were not suitable for any examination. A future experiment with a live monitoring which gives an online signal to the investigators in case of any abnormality in vital signs of the rats would give a more accurate data about the deceased rats during the study. The strength of our study is the absence of any confounding factors which might influence the observed oncocytic transformation. All rats were kept in the same standard laboratory environment and killed within the same daytime period in 2 consecutive days to eliminate the circadian rhythm differences between the groups at the time of sacrifice.

In conclusion, we showed that NMO induced an oncocytic change in pancreas, thyroid, pituitary, and adrenal glands. To the best of our knowledge, no identified specific environmental risk factors that lead to an oncocytic transformation in endocrine glands have been reported previously. One reason for the lack of extensive research in this area may be that oncocytic lesions are often characterized as benign tumors, which may not be a priority for researchers. However, a few oncocytic malignant tumors such as oncocytic adrenocortical carcinoma, oncocytic intraductal carcinoma of pancreas, oncocytic differentiated, poorly differentiated and medullary thyroid carcinomas, have also been reported. Therefore, given the increasing prevalence of endocrine-disrupting chemicals in the environment, personal care products, manufactured goods and food sources, there is a need to advance our understanding of the pathological mechanisms underlying oncocytosis in endocrine organs.

## Data Availability

The data that support the findings of this study are available from the corresponding author upon reasonable request.
